# Osteoarthritis After Anterior Cruciate Ligament Reconstruction: A Systematic Review of Contributing Factors and Potential Treatments

**DOI:** 10.7759/cureus.71188

**Published:** 2024-10-10

**Authors:** Shane Charles, Nick Preston

**Affiliations:** 1 Sports and Exercise Medicine, University of South Wales, Wales, GBR; 2 Orthopaedics and Trauma, San Fernando General Hospital, San Fernando, TTO; 3 Department of Rehabilitation Medicine, The University of Leeds, Leeds, GBR

**Keywords:** anterior cruciate ligament (acl), anterior cruciate ligament (acl) reconstruction, orthopaedics surgery, orthopedic sports medicine, post-traumatic arthritis

## Abstract

Anterior cruciate ligament (ACL) injuries result in abnormal knee motion and long-term joint degradation. ACL reconstruction (ACLR) is done with the aim of restoring normal knee kinematics and slowing the joint degradation process. It does appear that this inevitably happens and can be impacted by a multitude of factors. The aim of this review was to examine the factors that influence the progression of osteoarthritis (OA) after ACLR and examine possible treatments that can aid in slowing that progression. A systematic review was conducted by searching all levels of evidence for all studies in English that assessed risk factors for developing OA after ACL reconstruction, had a minimum follow-up of 10 years, and used radiographical outcomes to measure the presence of OA. Studies on trial treatments to reduce osteoarthritis after ACL reconstruction were also included. It was found that among the factors associated with an increased risk of post-ACLR OA are meniscal lesions, meniscectomy, increased age at the time of ACLR, increased time from injury to surgery, male sex, reduced range of motion, smaller thigh girth, graft complications, and failure. Additionally, in performing the ACLR, anteromedial femoral tunnel placement, higher graft tension, and following guidelines for performing anatomic ACLR have been shown to reduce the risk of OA as well. Patients should be adequately counselled on their risk pre-operatively for informed decision-making. Surgeons should also be aware of potential risk factors and how they can be mitigated.

## Introduction and background

Aims/hypothesis

The aims of this review were to determine the factors that impact the development of tibiofemoral osteoarthritis (OA) after surgical reconstruction of the anterior cruciate ligament (ACL) injuries in adults and to investigate what interventions there are to mitigate the development of osteoarthritis after reconstruction surgery for anterior cruciate ligament injuries in adults.

Background/introduction

The ACL is one of the most commonly injured ligaments in the knee. It accounts for approximately half of all knee ligament injuries [1]. Suffering an ACL tear can be severely damaging to an athlete’s career, as it can have them sidelined for several months and even limit their future ability to participate in cutting sports, though approximately 83% of high-level athletes do return to sport after injury [2]. The impact of this injury on the general population, with persons aiming to maintain activity for their well-being, is difficult to quantify. ACL tears can be treated conservatively or via surgical methods, including repair and reconstruction. Surgical reconstruction aims to restore the knee’s natural biomechanics and kinematics, which become impaired in the ACL-deficient knee [3]. However, it has been reported that approximately 12% of persons develop signs of OA within five years after ACL reconstruction (ACLR) [4], with 0.97% progressing to knee arthroplasty within 10 years [5]. The impact of osteoarthritis cannot be overstated. Patients suffer from pain, limitation in ability to conduct daily activities, and functional impairment. Surgery and rehabilitation are challenging even for the elite athlete, and the consequences of such injuries in the general population are difficult to capture. Given that ACL injuries primarily affect younger patient groups [3], a significant number of young individuals are at an increased risk of developing post-traumatic arthritis, even after undergoing reconstruction. Bearing this in mind, patients choosing to undergo ACLR should be knowledgeable of their risk of progression to OA. This knowledge will also help in future determinations of which patients are most likely to benefit from ACLR and the development of an optimal standardized pathway for the management of ACL injuries, as well as in determining how best personally tailored treatments can be provided. This is especially important for non-elite athletes who need not perform at a high level but do not want to face the challenges posed by early-onset OA. While previous studies have primarily focused on the development of post-traumatic arthritis in patients with conservatively managed ACL injuries, there is a dearth of data specifically examining the development of OA in patients who have already experienced ACLR and the factors that influence its progression. Therefore, this study aims to determine the factors that impact the development of tibiofemoral OA after ACLR and to look at the treatments available to mitigate the progression of osteoarthritis after ACLR in adults.

## Review

Methodology

Study Search and Selection

The study search process began with searching the EMBASE, CINAHL, DARE, and Cochrane databases. Additionally, Google Scholar and the University of South Wales Library were searched for additional studies to identify any possible grey literature relevant to the topic. The search was conducted using the terms “Arthritis” OR “post-traumatic arthritis” OR “Osteoarthritis” OR “post-surgical arthritis” OR “OA” AND “anterior cruciate ligament” OR “ACL” AND “Reconstruction” OR “Surgery” OR “repair” OR “ACLR.“ Study titles were examined for relevance and progressed to abstract screening once suitable, where they were screened according to the inclusion/exclusion criteria. The Covidence Systematic Review Software/Program was used to manage the workflow and automate the removal of duplicates. Due to the nature of this intervention, there were some limitations in finding studies that assessed the outcomes prospectively and had suitable control groups or participants without other confounding factors. This is so because ACL injuries are commonly associated with other lesions within the knee joint, all of which can impact outcomes.

The papers included had to be systematic reviews, meta-analyses, randomized control trials, case-control, cohort, or high-quality retrospective studies, published between the years 2005 and 2024, had to assess risk factors for developing OA after ACL reconstruction, must have had a minimum follow-up of 10 years, must have been in English, and must have used a radiographical outcome measure to assess the presence of OA.

Studies on the impact of knee biomechanics and kinematics on the development of arthritis after ACL reconstruction, studies assessing radiographical impact on cartilage after ACL reconstruction, and studies on available treatments and trial treatments to reduce osteoarthritis after ACL reconstruction were included to answer the review questions adequately. Studies on revision ACL surgery, those done on animal models, or those with follow-ups of less than 10 years were excluded. Those studies solely on conservatively managed (no surgery) ACL injuries were excluded. Studies on patellofemoral OA, those that defined OA symptoms using questionnaires or surveys without radiographic evidence, were excluded. Studies that met the eligibility criteria for the review had their entire manuscripts analysed and assessed according to the Critical Appraisal Skills Programme (CASP) protocols to assess their extrinsic and intrinsic quality and bias risk. Issues that arose regarding study selection and data extraction were handled by consultation with a specialist in the field, as well as the tutor for this project.

Data Management

Data extracted from the studies included general information (e.g., author, article title), study characteristics (e.g., aim/objectives of the study, study design, study inclusion, and exclusion criteria), participant characteristics (e.g., demographics, co-morbidities), data on the intervention, the radiological outcome measure used, statistical techniques used, length of follow-up, etc. A systematic analysis was conducted on the assessed studies. This involved an analysis of the commonly emerging themes seen between the studies to determine which risk factors may contribute to the development of osteoarthritis after ACLR, with any odds/hazard ratios reported. The studies and their characteristics and findings were tabulated for visual representation and ease of comparison. Due to the significant heterogeneity between the studies, this precluded us from doing a meta-analysis of the reviews included, and a systematic review was therefore done.

Ethical Approval

Ethical approval was sought and granted by the Ethics Review Board of the University of South Wales. This was deemed to be a low-risk study as it involved no patient-identifiable data and did not influence the course of patient treatment.

Disclaimer

This work was completed in partial fulfilment of an MSc in Sports and Exercise Medicine from the University of South Wales.

Results

**Figure 1:**
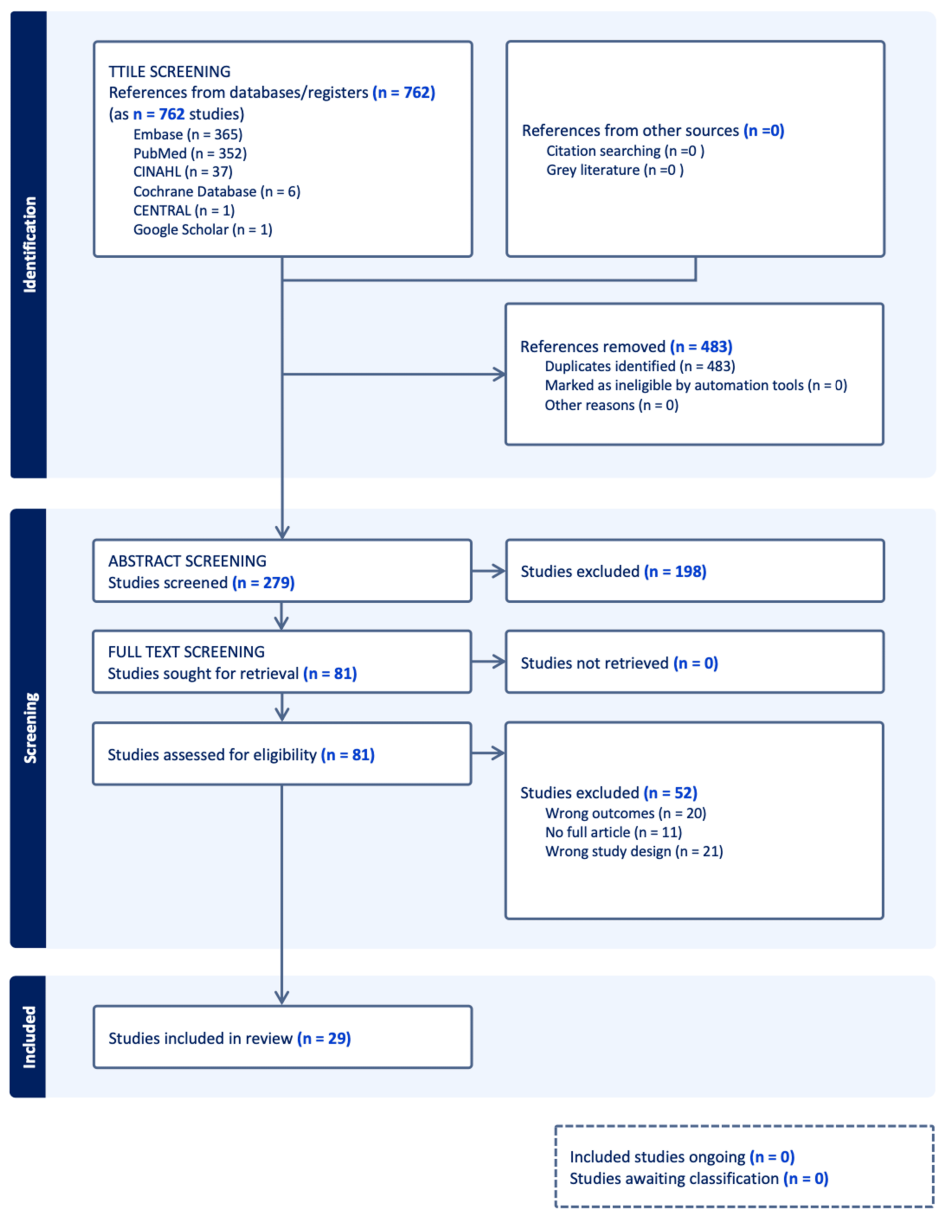
PRISMA chart

The above-mentioned search criteria yielded over 8000 titles from several databases. A single author highlighted 762 titles for formal title screening and selected 279 for abstract screening. A single author advanced 81 of those studies to full-text screening, resulting in the inclusion of 29 in the final analysis and the extraction of their data. This is highlighted in Figure 1. Data extraction was done according to the fields highlighted in the methodology and put into a templated data extraction spreadsheet in Appendix Table 1.

In total, 29 studies were included in this analysis. There was a significant level of heterogeneity among the studies. Their structures ranged from case series to retrospectives and prospective cohorts to meta-analyses. There was also significant heterogeneity among the studies in terms of the number of participants, methods used, and units of assessments of outcomes used.

The factors influencing the progression of osteoarthritis after ACLR were examined. It is best to interpret the results in a systematic manner. Therefore, we will look at pre-surgical, surgical, and post-surgical factors.

Pre-surgical Factors

Meniscal lesions: Meniscal lesions found in the preoperative MRI were found to impact the development of OA post-ACLR in our review. This was seen by Barenius et al. [6], who, in a study of 164 patients with long-term follow-up, found an increased risk of OA development in persons who had medial meniscus lesions at the inclusion of the study (OR: 2.5 (95% CI: 1.23-5.24), p = 0.011). Notably, Filbay et al. [7], in a study that included 234 patients at very long-term (32-37 years post-ACLR) follow-up, found that a baseline meniscal lesion increased the odds of developing osteoarthritis by three times (OR: 3.2 (95% CI: 1.3 to 8.3)). Costa-Paz et al. [8] found that patients with meniscal lesions had nearly four times (OR: 3.96, p = 0.039) increased odds of OA developing after ACLR. Even though the study by Seon et al. [9] had a small sample size of 58 patients, a meniscal lesion was associated with a greatly increased risk (OR: 9.19, p < 0.001). Furthermore, in a study by Øiestad et al. that included 181 participants, it was seen that the ACL rupture combined with the meniscal injury group had a higher prevalence of knee OA compared with the isolated ACL injury group [10].

Sex: Generally, as seen in a multicentre study by Curado et al. [11] with 182 patients (67 of them being male), male sex appears to confer an increased risk (p = 0.00018). This was confirmed by a study by Shelbourne et al. [12], where it was seen that male sex resulted in an increased odds (OR: 2.38 (95% CI: 1.44-3.94), P = 0.0007) of developing moderate to severe OA.

Age at surgery: In our review, it was found that increased age at the time of ACLR was associated with an increased risk of OA. This was seen in several studies, including Shelbourne et al. [12], where increased age conferred an odds ratio of 1.06 (1.03-1.09), P < 0.0001 for increasing OA risk. Curado et al. [11] showed that age >30 was associated with increased risk (p = 0.0026). Age >25 at ACLR was shown to have increased risk in the study by Seon et al. [9], with a nearly four times increased risk (OR: 3.365, p = 0.034). Lindanger et al. [13] found that patients with a Kellgren-Lawrence rating greater than 2 were, on average, slightly older as well (OR: 1.046 (95% CI: 1.005-1.088), P = 0.023).

BMI: Curado et al. [11] found that BMI was not associated with an increased risk of post-ACLR OA. However, only 2% of the patients in the study were obese; no comment on the statistical significance of the result was made, and thus, it would be difficult to form a conclusion on the relationship from the study. However, Barenius et al. [6] found BMI to be a risk factor. In this study, 164 patients were randomized to receive either a PT graft or an ST graft, and outcomes were assessed using the Kellgren-Lawrence classification. After adjusting for medial meniscal resection, it was found that BMI resulted in an increased risk of OA for the medial compartment (OR: 3.1 (95% CI: 1.22-7.89) P = 0.004), as well as for the patellofemoral joint (OR: 3.5 (95% CI: 1.53-7.84) P = 0.004).

Time from injury to surgery: In several studies in this review, it was seen that the time from initial injury to having ACLR impacted OA outcomes. Curado et al. [11] found that those who waited more than 16 months to have surgery after injury had a higher risk of OA (p = 0.0041). In a study by Sanders et al. [14], early surgery was defined as ACLR within one year of injury and delayed surgery as more than one year after injury. It was found that those who delayed surgery had a four times increased risk of developing a meniscal tear (HR of 3.9 (95% CI: 2.2-6.9)) and a six times higher risk of developing OA (HR 6.2 (95% CI: 3.4-11.4)). Of note, the study by Seon et al. [9] found that even delaying ACLR six months after injury increased the chances of developing OA (OR: 4.767, p = 0.021). Only the study by Barenius et al. [6] reported that time from injury to surgery was not a risk factor in groups defined as surgery within six months or after, or within 12 months or after (OR: 1.0 (95% CI: 0.99-1.02), p = 0.45).

Surgical Factors

Meniscectomy: Meniscectomy has been shown to be independently associated with increased risk (p < 0.05) of OA post-ACLR [12], and this association has been seen in several studies. For example, in meta-analyses done by Ruano et al. [15] and Claes et al. [16], both that included 1554 patients across all studies, meniscectomy was likely to increase the risk of developing OA by 3.54 times (95% CI: 2.56-4.91). Even though there was significant heterogeneity between the studies, the combined estimate for the prevalence of OA in the group without meniscectomy was 16.4% (95% CI: 7.0-33.9), compared to 50.4% (95% CI: 27.4-73.1) in the group with meniscectomy. Nakagawa et al. [17] showed that meniscectomy increased the odds (OR: 34.1, 95% CI: 2.2-522.4, p = 0.01), with no significant difference if it was medial or lateral meniscectomy (p = 0.17). Lindanger et al. [13] also showed the same. Medial meniscectomy increased the risk (OR: 1.876 (95% CI: 1.026-3.431), p = 0.041), as well as lateral meniscectomy (OR: 1.960 (95% CI: 1.048-3.669), p = 0.035). Shelbourne et al. [12] had similar results, with patients with medial meniscectomy having increased odds ratios of 1.8 (95% CI: 1.4-2.5, p = 0.0012) and 1.1 (95% CI: 0.85-1.7, p = 294) for lateral meniscectomy. Interestingly, Barenius et al. [6] showed that meniscus repair (OR = 0.8) reduced the risk of OA compared to meniscectomy (OR = 4.2), making a case for meniscus preservation over removal.

Open versus arthroscopically assisted surgery: Holm et al. [18] conducted a randomized control trial with a sample size of 67 patients and found no difference (P = 1.000) in radiographic osteoarthritis outcomes between open and arthroscopically assisted surgery. However, this is quite a small sample size.

Graft type: D'Ambrosi et al. [19] found, in a study including over 1500 operated knees with 20 years of follow-up, that patients with ITB grafts had the worst OA outcomes, with 71% developing moderate to severe OA. Patients with bone-patellar tendon-bone (BPTB) grafts had more radiographic OA than those with HT grafts (29% compared to 13%). This, however, was not proven to be statistically significant. Holm et al. [20] showed that BPTB grafts had a higher rate of OA compared to HT grafts at 10-year follow-up (55% with mild to moderate OA compared to 64%, P = 0.27). Furthermore, Sanders et al. [14] found in a study that included 964 patients that the use of allograft tissue in ACLR was associated with an increased risk of developing arthritis (HR: 4.9 (95% CI: 2.05-11.65), P < 0.001).

Tunnel placement: A systematic review and meta-analysis by Cinque et al. [21] found that using the transtibial (TT) approach to bone tunnel placement was associated with a higher risk of developing OA when compared to the anteromedial (AM) approach. Compared to the anteromedial approach, the rates of post-traumatic osteoarthritis (PTOA) on long-term follow-up of 10 years for the transtibial and anteromedial approaches were 45.6% and 31.2%, respectively (p<0.0001).

Graft tension: In a randomized controlled trial conducted by Costa et al. [22], patients 10-12 years after surgery were allotted to either have their grafts inserted under high or low tension at the time of surgery. The level of tension was determined by either inserting the graft with the knee in 30° of flexion for the high-tension group or with the knee fully extended (zero degrees of flexion) for the low-tension group. OA outcomes were then assessed using the MRI WORM and radiographic OARSI score. Generally, it was found that there was no significant difference between the groups for the WORM (P = 0.374) and OARSI (P = 0.179) scores. However, it was seen that males appeared to have relatively poorer outcomes for the OARSI scores in the low-tension group (P = 0.006). This seems to suggest that males with low-tension grafts may be predisposed to having OA after ACLR. It is important to note, however, that this study was conducted with a relatively small group of patients (n = 85), and there was a variety of grafts used, introducing heterogeneity and bias.

Anatomic versus non-anatomic reconstruction: In a study by Rothrauff et al. [23], it was assessed whether doing an anatomic reconstruction would result in less risk of OA compared to a non-anatomic one. It was found that the anatomic group had an OA prevalence of 23.2%, and the non-anatomic group had an OA prevalence of 43.9%. Notably, the anatomic group on average checked 9.2 ± 1.3 of the anatomic ACL reconstruction checklist (AARSC) criteria as defined by van Eck et al. [24], and the non-anatomic group 5.1 ± 1.1. An anatomic ACLR was therefore associated with a lower risk of post-ACLR OA.

Post-surgical Factors

Range of motion loss: This review showed that ROM after surgery was a prognostic factor in the progression of OA. Shelbourne et al. [25] found that loss of any knee extension at long-term follow-up was associated with an increased risk of OA development. Loss of knee extension (OR: 3.86 (95% CI: 2.38-6.26), P < 0.0001) and loss of knee flexion (OR: 3.36 (95% CI: 2.14-5.27), P < 0.0001) increased the likelihood of having OA at 20-year follow-up. It is therefore necessary to aim to achieve full knee ROM at discharge from physiotherapy, as not achieving this goal increases the risk of knee extension loss (OR: 19.7 (95% CI: 10.59-36.65); P <0.001) and flexion loss (OR: 7.97 (95% CI: 4.96-12.86), P < 0.001).

Quadriceps Strength

Øiestad et al. [26] found that loss of quadriceps strength over time was associated with an increased risk of OA (OR: 1.00, 95% CI: 1.00-1.01). This, however, is a very minuscule increase in risk as seen by the OR of 1. Zandiyeh et al. [27] however, in a study that assessed the differences in symmetry of muscle activation more than 10 years after ACLR, found that on MRI assessment via the WORMS score, patients with smaller thigh muscle girth had more signs of degenerative OA on long term follow up (p = 0.009). This was, however, a quite small study of 11 cases and 12 controls for comparison.

Return to Activity

Øiestad et al. [28] assessed the impact of returning to pivoting sport on the progression of OA after ACLR. 71% of patients in that study returned to sport post-ACLR. Of that population, 51% were able to return to their previous sport, with 18.5% of them having radiographic OA at long-term follow-up. Despite this, only 5.5% of the return to sport cohort had symptomatic OA as determined by KOOS scales. In the group that did not return to pivoting sports, 42% had radiographic OA, and 25% had symptomatic OA. It was seen, therefore, that return to pivoting sport post-ACLR was associated with a reduction in OA risk (OR: 0.40, 95% CI: 0.17-0.98) at 15-year long-term follow-up. A similar result was also seen in a study by Lindanger et al. [13], where a return to preinjury level of sports was associated with a reduced risk of developing OA (P = 0.062).

Graft Rupture

In a study by Söderman et al. [29], KL classification was used to determine OA, and MRI was used to determine the integrity of the graft at long-term follow-up of 30 years post-ACLR. Patients with ruptured and missing grafts were found to have worse outcome scores and increased signs of OA in the tibiofemoral and patellofemoral compartments (p = 0.0003). This displays the impact of the ACLR on restoring stable ligamentous structures in the joint and its ability to reduce the risk of progression to OA.

Residual Laxity

Struewer et al. [30] conducted a retrospective analysis of hamstring grafts with long-term follow-up. The study did suffer from significant loss to follow-up; however, it did show that OA progression was associated with decreased stability of the graft and joint over time (p < 0.05). This was also seen by Curado et al. [11], who demonstrated that residual laxity of the joint >5 mm after ACLR was associated with an increased risk of OA (p < 0.05).

**Figure 2:**
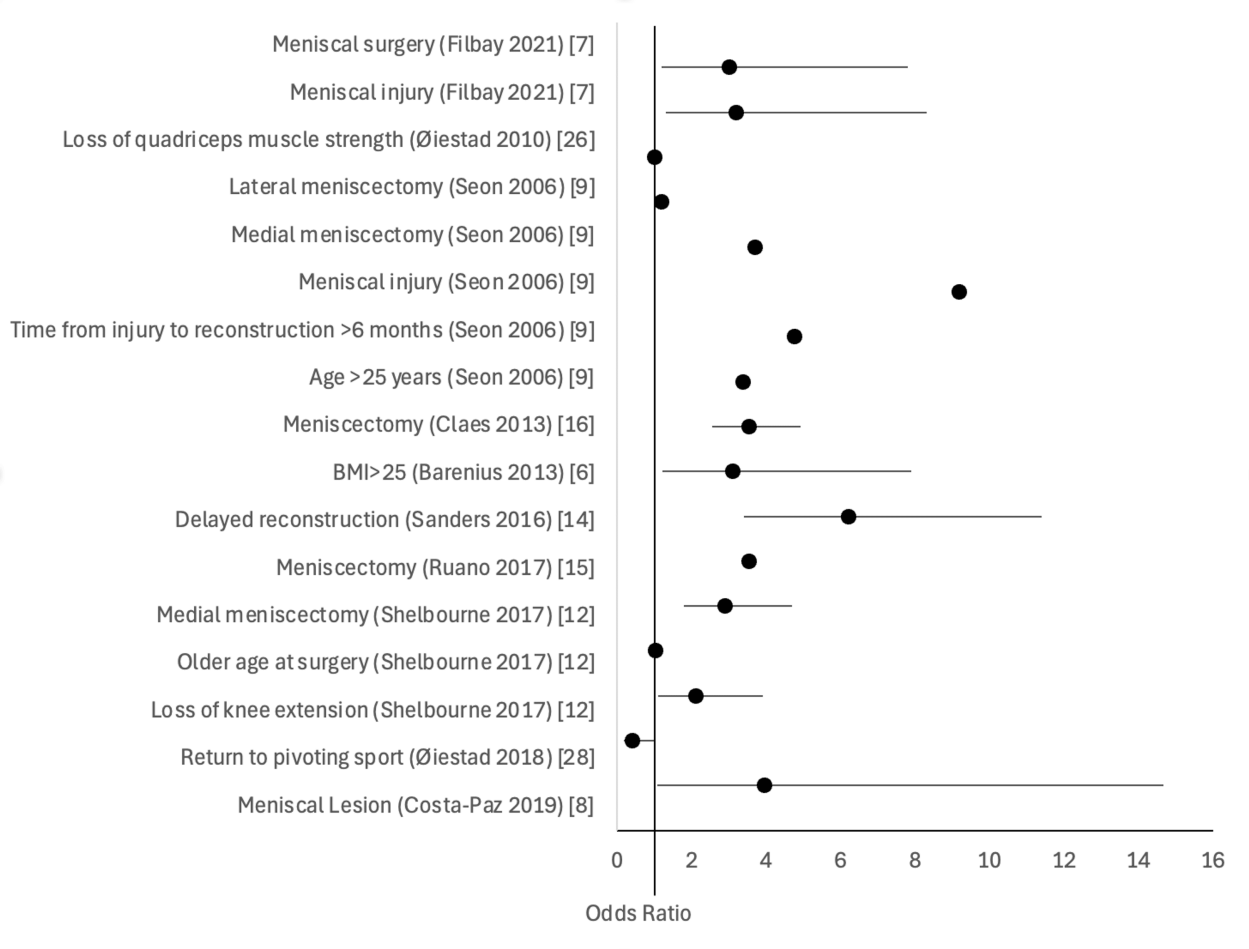
Plot of effect sizes mentioned in included studies

A summary of all the studies that mentioned the effect sizes of the analyzed factors can be seen in Figure 2.

Possible Treatments

Options for the treatment of OA post-ACLR are being discovered in a new region of medicine. There are, however, some available options on the market. For example, in a randomized controlled trial with 75 patients, Blackburn et al. [31] allocated patients to either receive whole body (WBV) or local muscle (LMV) vibratory stimuli with the aim of improving neuromuscular control post-ACLR. For the whole-body group, this involved using a specific machine to apply a vibratory stimulus to the entire body to cause repeated flexion and extension stimuli to the joints of the lower extremities. The local muscle device caused a similar response but was attached instead to one muscle (e.g., the quadriceps muscle). It was found that WBV increased the peak internal knee extension and that LMV improved the loading rates of the joint in gait. Both of these tenets of the gait cycle were associated with PTOA development after ACLR [31]. 

Further, Andriolo et al. [32] conducted a systematic review of the use of platelet-rich plasma (PRP) in the ACLR population. In that study, it was seen that PRP can be used at many steps along the ACLR process, including in the harvest site for healing, intra-articularly at the end of the surgery and in the bone tunnels. In the final analysis, the use of PRP had mixed results but showed itself to be a safe option, with potential benefits for graft harvest site healing and graft maturation. No significant benefit was seen for graft integration. Additionally, Kon et al. [33] also conducted a systematic review of treatments for post-ACLR OA, including uses of PRP and stem cells. In that review, two studies showed no benefit in the use of either adipose-derived stem cells (ADSCs) or bone marrow concentrate (BMC), respectively, when they were integrated into the ACLR procedure. Regarding the use of PRP, several papers in the review reported positive benefits of PRP for graft maturation, reducing post-op stiffness and improving graft stability.

Discussion

ACL injuries are common. They affect approximately 1 in 3500 people in the United States, and there are around 400,000 ACLRs done yearly [3]. ACL tears account for approximately half of all knee ligament injuries. These injuries can happen to anyone, but athletes participating in pivoting sports, as well as female athletes, are at greater risk of suffering an ACL tear [34]. An ACL injury can have an athlete sidelined for months. Approximately 83% of high-level athletes can return to sport after injury [2]. Female athletes are also especially susceptible to ACL injury [34]. This is thought to be due to anatomic factors like their smaller femoral intercondylar notch and pelvic Q angle, as well as hormonal factors that seem to impact ligamentous laxity [34,35]. The average age at which one suffers an ACL injury is 33.9 years [36].

There are several ways in which an ACL tear can be managed. Conservative management mandates aggressive physiotherapy to regain similar pre-morbid function, and the patient-reported outcomes have been shown to be similar to that of surgical management [37]. However, patients selecting this treatment option must be aware of the implications of the ACL-deficient knee and its impact on the meniscus [34], which works to prevent aberrant force transmission in the joint and plays a pivotal role in the prevention of osteoarthritis. 

It was seen that the status of the meniscus is one of the greatest prognostic indicators. Loss of meniscal substance and meniscal tears results in abnormal force distribution and degenerative changes in the joint over time, accelerating the OA process. Acute tears, those smaller than 2 cm, and vertical longitudinal tears increase the chances of a meniscal repair healing and tears in the vascularised outer zone [36]. As much as possible, surgeons should attempt to preserve the meniscus, as without it, as seen in this review, patients are predisposed to pain and early-onset OA [38,39].

As mentioned earlier, females are at higher risk of developing ACL tears due to structural and hormonal factors. From the review, however, it was males who seemed to have a higher risk of OA progression after ACLR. The results have been mixed, though. Against this background, it is important to note that males were more likely to return to sport and have higher activity levels than females after ACLR [40].

It was seen that increased age at the time of ACLR significantly increased the risk of OA post-ACLR. Due to the high heterogeneity between the studies, however, it is difficult to quantify how the level of risk increases with age at the time of ACLR. This, therefore, makes it difficult to possibly determine a cut-off age, after which the risk of doing an ACLR to slow the progression of OA may outweigh the benefits. What is partially unclear as well is whether patients being older at the time of surgery is also associated with more chronic ACL tears, and there may, therefore, be higher levels of OA already present in the knee, thereby acting as a confounding factor. Older patients must therefore be adequately counselled before undergoing ACLR on the risks, including their long-term risk of OA progression.

The time from injury to surgery also significantly influenced disease progression. The impact of an ACL-deficient knee on OA progression has been highlighted above. The question then becomes, What is ideal timing? How early is too early, and how late is too late? In a paper by Evans et al. [41], several systematic reviews on the topic were investigated to determine an answer. It was found that surgery too early (before four to six weeks) can be complicated by arthrofibrosis, and surgery after 5 months but before 24 months was associated with worse subjective outcome scores than those who had early surgery. Furthermore, our study showed that surgery later than six months after injury increased the risk of developing OA. It would, therefore, appear that the optimal timing would be somewhere within that window. While the heterogeneity of the studies may have made it difficult to ascertain a golden or specific time frame within which the risk of OA progression is minimized, the general trend was that earlier surgery was better.

The influence of BMI remains contentious. Further studies must be done to determine if it is a significant contributor. It would seem, though, that as with regular osteoarthritis, a higher BMI would result in a higher OA risk [42]. However, this association remains to be determined clearly in the post-ACLR population.

Regarding surgical technique, it was seen that an anatomical reconstruction was superior to a non-anatomical reconstruction for reducing OA risk. The definition of an anatomic ACLR was based on the Anatomic AARSC developed by van Eck et al. [24]. The AARSC was also shown in a study by Samuelsson et al. [43] to have a lower risk of revision surgery compared with trans-portal drilling with anatomic tunnel placement. Moreover, as seen by Rothrauff et al. [23], using the AARSC also reduced the risk of developing OA post-ACLR. The AARSC is, therefore, a suitable guide for surgeons doing ACLR to follow.

As seen in this review, arthroscopic surgery had no additional benefit compared to open mini arthrotomy when examining OA outcomes [16]. Arthroscopic surgery does, however, have the benefits of smaller incisions, less post-operative pain, and a shorter length of stay [44], and it would be understandable why patients would opt for that method over open surgery.

Regarding OA outcomes, it was found in this review that there was no significant difference in the risk of developing OA post-ACLR between the different types of autografts, with the main ones used including PT and HT grafts. The choice of graft, therefore, comes down to surgeon and patient preference and which pros and cons they are willing to accept. On the other hand, however, autografts generally have been shown to have better outcomes than allografts, as allografts increase the risk of OA as well as of graft rejection, have a higher rupture risk, and have less favourable patient-reported outcomes [45]. 

During an ACLR, once the type of graft has been selected, one has to determine how they are going to place the graft. The main options for femoral tunnel drilling and placement are the TT, AM, and outside-in (OI) techniques. There are advantages and disadvantages to each technique. However, the TT technique has been associated with longer and more oblique femoral tunnels, possibly resulting in the AM technique giving better stability and postoperative outcomes than the TT technique [46]. In a two-year follow-up study by Carllee et al. [47], it was found that there was no significant difference in graft rupture, outcome scores, and function between the OI and AM techniques. Furthermore, our review found that the TT approach was associated with a higher risk of developing OA post-ACLR than the AM approach [19]. It has been suggested that the TT approach may be less anatomic and thus allow for more abnormal rotation and anterior translation of the joint compared to the anteromedial approach [48].

Lastly, early and aggressive physiotherapy is the cornerstone of recovery from ACLR. It is just as important as the surgery itself in getting patients back on their feet and improving their outcomes [49]. Persons who suffer ACL injuries are often young, active individuals who participate in pivoting/cutting sports. These injuries are big blows to their usual activity levels, and they often want to return to sport as soon as possible. In this review, it was seen that a return to pivoting sport was associated with a reduction in the risk of developing OA post-ACLR [28]. It can be that those patients with less symptomatic and radiographic OA progressed well to return to sport, while the others did not, thereby skewing the result. However, as it stands, it appears that a return to pivoting sports may be a protective factor against OA.

Patients with ACL injuries have abnormal gait. Analysis of their gait displays reduced peak knee flexion angles and sagittal plane moments. These have been shown to be associated with poor quadriceps function [31]. As seen in the study by Blackburn et al. [31], a vibratory stimulus was shown to improve quadriceps muscle function and improve gait biomechanics associated with post-traumatic osteoarthritis. This was done by either the use of a whole body or a local muscle vibration machine. Both of these stimuli were shown to improve aspects of the gait cycle. This is, therefore, a method that can be integrated into the physiotherapy regimen. Furthermore, PRP was shown to have some benefits when integrated into ACLR. In the systematic reviews by Andriolo et al. [32] and Kon et al. [33], it was shown that PRP can be used at many of the ACLR steps, including in the harvest site for healing, intraarticularly at the end of the surgery, and in the bone tunnels. The integration of PRP was shown to have potential benefits for graft harvest site healing and graft maturation, as well as reducing post-op stiffness and improving graft stability.

There were several limitations in this study. As early ACLR surgery was routinely done using BPTB grafts, many of the studies assess outcomes related to those grafts. There remains to be a significant body of long-term data on OA outcomes in HT grafts and allografts. No studies assessed the impact of ethnicity or race on the progression of OA after ACLR. A lot of the studies included were based on majority Caucasian/European populations. It would be interesting to see how this factor can impact, especially given that some African and Indian populations have greater articular mobility [50]. Furthermore, this review was an assessment of radiological OA and not symptomatic OA. It was seen that not all patients with radiographic features of OA are symptomatic [18]. There was significant heterogeneity between the methods used in the studies. This included the types of grafts used, the surgical methods, the units of measurement of radiographic OA, and the time from injury to surgery. Additionally, there was also a significant difference between the study methods, as some studies were prospective while others were retrospective.

Though there was significant heterogeneity, it also contributed to the benefits of this analysis, as this review was able to capture a scoping view of the topic from many perspectives and through many methods over a long period of time.

Bias

Due to the heterogeneity between the study methods, participants, and outcome measures used, there was certainly some elevated risk of bias in this review. Most of the studies were also found in online databases, resulting in some measure of publication bias as well. Additionally, only English studies were included, introducing language bias into the review. Finally, some of the studies did not report on the size of the effects measured with confidence intervals or p-values. This made it difficult to know whether some results were truly significant or not. To mitigate the risks highlighted, the search for studies was significantly robust to ensure that all appropriate studies on the topic were included. Furthermore, significant attention was paid to ensure the studies selected for analysis strictly fit the eligibility criteria and were of high quality.

Recommendations

In the study by Rothrauff et al. [23], it was seen that an anatomic ACLR was associated with a decreased risk of OA. It also guides adequate documentation of the procedure and has been shown to have benefits for patients. It was also seen that deficiencies in quadriceps strength as well as limited range of motion after ACLR, can be deleterious to patients. All patients should, therefore, undergo early and tailored physiotherapy with the aim of achieving full range of motion in flexion and extension and developing adequate quadriceps muscle mass by the point of discharge from physiotherapy. Though only the study by Costa et al. [22] explicitly investigated the impact of tension on the ACL graft, there was some evidence that low-tension grafts should be avoided. Due to the potential benefit of returning to sport for OA outcomes, it can be encouraged but should be guided by professionals in the field. Early surgery is better. Several studies showed that delay of surgery was associated with poorer OA outcomes. ACLR should, therefore, be done as early as safely possible, as the risk of OA was shown to increase if surgery was done even at six months post-injury. Surgeons should consider persevering the meniscus as much as possible to reduce OA risk. This would involve attempting repair rather than heading straight to removal for meniscal injuries seen intra-operatively [51,52]. Surgeons can also give thought to using PRP during ACLR, as positive results have been shown thus far for graft maturation, stability, and donor site healing.

Further research

Though some studies did partially assess it, the outcomes of meniscectomy versus repair of meniscal tears and their impact on the development of OA deserve a dedicated study with a sufficiently large sample size. Biologics are also being investigated as a potential mediator of PTOA after ACLR. However, these outcomes have to be assessed with radiographic outcomes after long-term follow-up >10 years.

## Conclusions

This review showed that there are a multitude of factors that can impact the progression of OA after ACLR. Among the factors associated with an increased risk of post-ACLR OA include preoperatively diagnosed meniscal lesions, meniscectomy, increased age at the time of ACLR, increased time from injury to surgery, male sex, loss of knee flexion and extension, smaller thigh girth, graft rupture, and residual laxity of the graft. Meniscal injury, delayed reconstruction, meniscectomy, and older age at surgery seemed to have the greatest impact on post-ACLR OA and are the most well-documented influential factors. Additionally, in performing the ACLR, anteromedial femoral tunnel placement, higher graft tension, and following guidelines for performing anatomic ACLR have been shown to reduce the risk of OA as well. Finally, the importance of aggressive physiotherapy was seen as any deficit in the range of motion in flexion and extension was shown to impact OA outcomes. Deficits in quadriceps strength were also shown to contribute to abnormal knee kinematics, which can lead to increased risk. Further to this, it was seen that vibratory stimuli were able to reduce some of the quadriceps dysfunction associated with ACL injury and work as a useful adjunct in rehabilitation. PRP was also shown to be a useful aid, with its ability to be integrated and show benefit at multiple steps in ACLR. Patients should be adequately counselled on their risk pre-operatively for informed decision-making. Surgeons should also be aware of potential risk factors and the steps they can take to mitigate those risks.

## Appendices

**Table 1:** Summary table of included studies

Study ID	Methods: design	Sample size at follow-up	Length of follow up	Risk factor/treatment/intervention assessed	Outcome measures used	Main result
Barenius et al. [6]	Randomized controlled trial	135	14 years	Patellar tendon vs. semitedinosus grafts	KL. Patellar height: Insall Salvati index	No significant difference was seen between the graft types (P = 0.073). Risk Factors for Osteoarthritis were: BMI 25 kg/m^2^ at 2-year follow-up (OR 3.1 (1.22-7.89) p = 0.004) and Medial meniscus resection (OR 3.6 (1.42-9.29) P = 0.001).
Filbay et al. [7]	Prospective cohort study	251	32 to 37 years	Early surgical repair vs non-augmented repair plus physiotherapist-supervised rehabilitation vs physiotherapy only	Kellgren and Lawrence (KL) classification system	A preoperative meniscal injury increased the odds of OA (OR: 3.2 (95% CI: 1.3 to 8.3)), compared to no meniscal injury. Meniscal surgery resulted in a higher risk of OA (OR 3.0 (95% CI 1.2, 7.8)) compared to no meniscal injury or surgery.
Costa-Paz et al. [8]	Retrospective cohort study	72	Median follow-up of 22 (IQR 21–25)	Long-term ACLR follow up	IKDC criteria	Meniscal lesions were associated with an increased risk of developing OA (OR 3.96, 95% CI 1.07-14.65).
Seon et al. [9]	Prospective cohort study	58	11.2 years (range 8.6–13.8 years)	Long-term ACLR follow up, Patellar tendon autograft	Kellgren and Lawrence’s classification	Factors associated with increased OA risk: Age >25 years at the time of reconstruction - OR 3.37, p = 0.034. Time from injury to reconstruction >6 months OR 4.77, p = 0.021. Meniscal injury OR 9.19, p < 0.001.Medial meniscectomy OR 3.71, p = 0.03), and lateral meniscectomy OR 1.20, p > 0.05.
Øiestad et al. [10]	Prospective cohort study	181	10-15-years	Bone-patellar tendon-bone (BPTB) autograft, either with miniopen or arthroscopic procedure. Isolated vs combined ACLR and meniscal lesions	Kellgren and Lawrence (KL) classification system	Combined injury group resulted in significantly higher prevalence of radiographic knee OA compared with the isolated injury group (80% and 62%, P < 0.008).
Curado et al. [11]	Retrospective cohort study	182	22 ± 1 years	Long-term ACLR follow up	IKDC criteria	Risk factors for OA after ACLR: Age > 30 years at surgery (p = 0.0026), Male sex (p = 0.00018), Injury-to-surgery time > 16 months (p = 0.0041), Pivot sport (p ≤ 0.05), Moderate or strenuous physical activity (p ≤ 0.05), Meniscectomy (p ≤ 0.05), Residual laxity >5 mm (p ≤ 0.05).
Shelbourne et al. [12]	Retrospective cohort study	423	22.5 years (range, 20.0-33.1 years)	Long-term ACLR follow up, patella tendon autografts	IKDC criteria	Factors associated with increased OA risk after regression model include medial meniscectomy OR 2.9 (1.8-4.7) p ≤ 0.0001 loss of knee extension or p=0.024, older age at surgery.
Lindanger et al. [13]	Prospective cohort study	235	Median 25 years (range, 20-31)	Long-term ACLR follow up	Kellgren and Lawrence (KL) classification system	Medial and lateral meniscal surgery were found to be independent risk factors for OA development after ACLR (OR 1.88 (95% CI, 1.03-3.43; P = 0.041 for medial meniscal surgery and OR 1.96 (95% CI, 1.05-3.67; P = 0.035) for lateral meniscal surgery. Return to sports was associated with a decreased odds of developing OA (P = 0.06).
Sanders et al. [14]	Retrospective cohort study	964	13.7 years	Timing of ACLR, long-term ACLR follow up	Physician-diagnosed arthritis	Factors associated with increased risk of OA were: delayed reconstruction HR of 6.2 (95% CI, 3.4-11.4), age older than 21 years at the time of injury (P = 0.02), presence of both medial and lateral meniscal tears at the time of ACL injury (P < 0.001), treatment with meniscectomy (P = 0.07), an articular cartilage injury at the time of the ACL tear (P = 0.006) and use of allograft tissue during ACLR (P < 0.001).
Ruano et al. [15]	Systematic review	1554	10 to 24.5 years	ACLR with and without meniscectomy	Ahlba ¨ck, modified Fairbanks grades, KL and IKDC	Meniscectomy significantly increased the risk of developing OA by 3.54 times.
Claes et al. [16]	Meta analysis	1554	10 to 24.5 years	Long-term ACLR follow up	IKDC criteria	Meniscectomy was associated with a 3.54 times higher risk ((95% CI 2.56–4.91)) than ACLR without meniscectomy.
Nakagawa et al. [17]	Retrospective cohort study	40	Minimum 15 years	Long-term ACLR follow up	Kellgren and Lawrence (KL) classification system	Meniscectomy was identified as the only risk factor for OA progression (p＝0.01).
Holm et al. [18]	Randomized controlled trial	67	12 years	Endoscopic patellar tendon–bone (PTB) reconstruction (ENDO) vs open PTB reconstruction (OPEN)	Kellgren and Lawrence (KL) classification system	There were no significant differences between the OPEN and ENDO groups for OA outcomes, with 80% and 79% of the respective groups developing grade 2 or higher OA (P = 1.000).
D'Ambrosi et al. [19]	Systematic review	1552	Mean 23.34 ± 2.56 years	Long-term follow up of various ACLR grafts	Kellgren–Lawrence, IKDC and Ahlbäck classification.	71% of patients that received ITB graft were classified as IKDC C or D at radiographic follow‐up. For PT grafts (IKDC grades C and D—29% vs. 13%). No significant difference between the PT and HS grafts in terms of OA were seen. (19.3% for PT vs. 19.6% for HT, p = 0.94).
Holm et al. [20]	Randomized controlled trial	57	10 years	Patellar tendon vs. 4 bundle Hamstring graft	Kellgren and Lawrence (KL) classification system	No significant differences were found between the two types of grafts. 55% and 64% of the participants had significant OA in the hamstring and the PT groups, respectively (P = 0.27).
Cinque et al. [21]	Systematic Review	1546	10.9 years (5.4-17.8)	Anteromedial vs. transtibial approach to femoral tunnel positioning	Kellgren-Lawrence (KL) classification, Ahlback, International Knee Documentation Committee (IKDC), and Osteoarthritis Research Society International (OARSI).	TT approach associated with increased risk of PTOA (45.6%) compared with the AM approach (31.2%) at long-term follow-up (Z = 8.58; P < 0.0001)
Costa et al. [22]	Randomized controlled trial	95	10 to 12 years	Graft tension	OARSI, Whole-Organ Magnetic Resonance Imaging Score (WORMS)	Males with low tension grafts had significantly worse outcomes compared to females (P = 0.006).
Rothrauff et al. [23]	Systematic review	2224	Mean 15.3-15.9 years	Anatomic vs. non-anatomic ACLR	OARSI, WORMS, Anatomic ACL Reconstruction Checklist (AARSC)	23.2% of patients with anatomic ACLR developed OA compared to 43.9% that had non-anatomic ACLR.
Shelbourne et al. [25]	Prospective cohort study	780	Mean of 10.5 6 4.2 years	Knee motion after ACLR	IKDC criteria	At final follow-up, loss of range of motion, those who had partial medial meniscectomy, and whose who suffered articular cartilage damage each had about 2 times the odds of having OA.
Øiestad et al. [26]	Prospective cohort study	258	10-15 years	Quadriceps muscle weakness	Muscle strength - cybex 6000. radiologic examination - Kellgren/Lawrence classification	Loss of quadriceps muscle strength showed no significant association with developing OA post-ACLR. OR 1.00, 95% CI 1.0-1.01. Increased age and meniscal injury were associated with higher OA risk (OR 1.06 and 2.05 respectively).
Zandiyeh et al. [27]	Case-control study	11	11.9 ± 1.3 years	ACLR and Neuromuscular function impact on OA	WORMS	Smaller muscle girth of the thigh was significantly associated with inferior WORM scores (B0 = −8.3, ß = −20.8, p = 0.009).
Øiestad et al. [28]	Prospective cohort study	210	15 years	Return to pivoting sport	Kellgren and Lawrence (KL) classification system	Participants that returned to pivoting sports were likely to develop radiographic OA (OR 0.40, 95%CI 0.17 to 0.98) compared with those who did not.
Söderman et al. [29]	Retrospective cohort study	60	31 years (range 28-33 years)	Graft rupture	KL, MRI was used to assess the menisci and the structural integrity of the ACL graft.	Ruptured ACL graft graft was associated with increased risk of medial tibiofemoral OA (p = 0.0003).
Struewer et al. [30]	Retrospective cohort study	52	Average 10.2 years (8-13 years)	Hamstring tendon grafts/anterior laxity	Jäger-Wirth classification	There was a significant correlation between instrumental anterior laxity and radiological OA (p < 0.05).
Blackburn et al. [31]	Randomized controlled trial	75	Not stated	Whole body and local muscle vibration	Gait speed, peak knee flexion angle, peak knee virus angle, peak vGRF, Linear vGFR loading rate, Instantaneous vGFR loading rate, KAM - internal knee abduction moment, KEM - internal knee extension moment	Vibration reduced pain, improved quadriceps function, and enhanced CNS excitability.
Andriolo et al. [32]	Systematic Review	Not stated	Not stated	PRP integration in ACLR	Variable	PRP showed positive effects on graft maturation, bone healing.
Kon et al. [33]	Systematic Review	Not stated	Not stated	Biologic Agent use in ACLR	Variable	No benefit seen of ADSCs, or BMC. PRP shown to have less swelling post op, better function post op, better graft maturation.
Hart et al. [51]	Case-control study	31	Mean 10 years (9 to 13)	Arthroscopic bone-patellar tendon-bone reconstruction without notch- plasty	Ahlback system	ACLR with partial meniscectomy resulted in greater degenerative changes than ACLR without meniscectomy (P < 0.05).
Gerhard et al. [52]	Retrospective cohort study	63	Mean 16 ± 1 years	Long-term ACLR follow up	Kellgren and Lawrence (KL) classification system	Those with a meniscal lesion at the time of ACL tear showed less favourable outcomes than those with isolated tears (p < 0.05).
